# Perspectives of People Who Are Overweight and Obese on Using Wearable Technology for Weight Management: Systematic Review

**DOI:** 10.2196/12651

**Published:** 2020-01-13

**Authors:** Ruiqi Hu, Michelle Helena van Velthoven, Edward Meinert

**Affiliations:** 1 Digitally Enabled PrevenTative Health Research Group Department of Paediatrics University of Oxford Oxford United Kingdom; 2 Department of Primary Care and Public Health, School of Public Health Imperial College London London United Kingdom

**Keywords:** wearable electronic devices, wearable technology, wearable device, mobile health, digital technology, weight loss, wearable, activity tracker, obesity, overweight

## Abstract

**Background:**

Obesity is a large contributor to preventable chronic diseases and health care costs. The efficacy of wearable devices for weight management has been researched; however, there is limited knowledge on how these devices are perceived by users.

**Objective:**

This study aimed to review user perspectives on wearable technology for weight management in people who are overweight and obese.

**Methods:**

We searched the online databases Pubmed, Scopus, Embase, and the Cochrane library for literature published from 2008 onward. We included all types of studies using a wearable device for delivering weight-loss interventions in adults who are overweight or obese, and qualitative data were collected about participants' perspectives on the device. We performed a quality assessment using criteria relevant to different study types. The Cochrane risk of bias tool was used for randomized controlled trials. The Risk of Bias in Non-randomized Studies - of Interventions (ROBINS-I) was used for nonrandomized studies. The Oxman and Guyatt Criteria were used for systematic reviews. We used the critical appraisal checklist for qualitative studies. Data were extracted into a data extraction sheet and thematically analyzed.

**Results:**

We included 19 studies: 5 randomized controlled trials, 6 nonrandomized studies, 5 qualitative studies, and 3 reviews. Mixed perceptions existed for different constructs of wearable technologies, which reflects the differences in the suitability of wearable technology interventions for different individuals in different contexts. This also indicates that interventions were not often tailored to participants' motivations. In addition, very few wearable technology interventions included a thorough qualitative analysis of the participants' view on important features of the intervention that made it successful.

**Conclusions:**

This study highlights the importance of determining the type of intervention most suitable for an individual before the intervention is used. Our findings could help participants find a suitable intervention that is most effective for them. Further research needs to develop a user-centered tool for obtaining comprehensive user feedback.

**Trial Registration:**

PROSPERO CRD42018096932; https://www.crd.york.ac.uk/prospero/display_record.php?RecordID=96932

## Introduction

Obesity is an increasing but preventable chronic health problem affecting over half of the world’s adult population and costing the United States an estimated US $147-$210 billion per year [1]. Obesity is a complex issue involving various interacting factors including a person’s upbringing, lifestyle, environment, and genetics. Numerous strategies for losing weight have been developed over the past decades, which mainly focus on reducing calorie intake and increasing energy expenditure.

Ownership of smartphones has increased among every demographic group including low-income populations, accompanied by a rapidly growing wearable sector [2]. There are many technologies available that can facilitate the delivery of weight management interventions, for example, wearable devices and smartphone apps. In more recently developed products, the wearables and apps can be synced through Bluetooth for long-term data tracking [3]. In combination with an effective weight management intervention, technologies can help weight loss through various means, for example, by promoting physical exercise, monitoring food consumption, or encouraging interuser communication and support [4].

Several health behavior theories can be used to design more effective weight loss interventions, for example, the self-determination theory, social cognitive theory, and elaboration likelihood model. These theories suggest that behavioral change is based on an individual’s reception to things people encounter in their environment. A key aspect is whether the intervention is received favorably by the target audience [5]. It is important to understand what individuals who are overweight and obese value about wearable technology for delivering weight management interventions. This information can be used for the future design of efficient weight loss interventions assisted by wearable technology.

Studies have been conducted to assess the effectiveness of wearables or mobile apps to increase physical activity and decrease sedentary behavior [6]. However, few have investigated their efficacy in achieving desired health outcomes [7]. Even fewer have investigated how these devices are perceived by users [8].

This review aims to review user perspectives on wearable technology for weight management in people who are overweight and obese. Additionally, discrepancies in opinions of participants and investigators that may have contributed to this difference were reviewed. This review answers the following question: From a user feedback perspective, what makes wearable technology efficacious for weight management?

## Methods

### Protocol and Registration

A protocol was registered with PROSPERO (CRD42018096932), with the review structure following PRISMA (Preferred Reporting Items for Systematic Reviews and Meta-Analyses) guidelines (Multimedia Appendix 1).

Research issues identified and prioritized by members of the public in a workshop at the European Scientific Institute in July 2017 were used to guide the focus of this study. As data collection was executed via published literature, ethical approval was not required for this review.

### Eligibility Criteria

We included studies on participants who were obese or overweight and above the age of 18 years. “Overweight” was defined as having a body mass index of 25-29.99 kg/m^2^ or as defined by the study, and “Obese” was defined as having a body mass index of ≥30 kg/m^2^.

Interventions included were digital wearable technologies used for monitoring or managing weight. Devices needed to have a clear use case for these activities, and we included studies examining the effectiveness of these devices for this purpose.

Comparators included traditional behavioral weight loss approaches, usual care, another intervention, or no intervention. Studies that did not have a comparator were also included if they met the other inclusion criteria.

Outcomes were barriers and facilitators for management weight and factors influencing the design, development, and deployment of wearable technologies for delivering interventions.

We included all types of studies where qualitative data were collected about the participant’s feedback on the device. Documents written in English that were published after 2008 were included.

### Information Sources

We searched the electronic databases PubMed, Medline, Scopus, Embase, and the Cochrane Library from 2008 onward. Data prior to 2008 were not included because they did not consider the rapid change in the use of smartphone technology and its influence on the development of wearable technology.

### Search

A combination of keywords and index terms related to items in the PICO (Participants, Interventions, Comparators, Outcomes) approach were used to search for relevant papers. A librarian was consulted for advice on the searches. PubMed was the first database searched after a list of keywords and Medical Subject Headings terms were chosen. The search was then adjusted and modified for subsequent databases. Search strings for all databases can be found in Multimedia Appendix 2. In databases where index terms did not exist, keywords were used instead.

### Study Selection

Duplicate articles were first removed using EndNote X8 software (Clarivate Analytics, Philadelphia, PA), and then any remaining duplicates were deleted manually. Two further screens were completed using endnote according to the eligibility criteria: (1) any field contains “device*” or “mobile” or “track*” or “technolog*” or “electronic,” “not child*,” “not adolescent*,” and “weight*” and (2) abstract contains “overweight” or “obes*.” Following the second screen, the titles and abstracts of all remaining studies were screened individually by two reviewers. Any discrepancies between the two reviewers were resolved by discussion. A list of studies for full-text reading was produced. The inclusion and exclusion criteria were used to select relevant studies. A final list of eligible studies was created when ineligible studies were excluded following reading the full-text papers. Reference lists of included studies were searched, and no further eligible studies were identified.

### Data Collection Process and Items

A standardized data extraction sheet was used to extract data. The extracted data included the title, research question, data sources, how the data were analyzed, main findings, and conclusions. During the data extraction process, a reflection of the gathered evidence on the research question was also created.

### Assessment of Methodological Quality

All eligible studies in the final list underwent a methodological quality assessment. Different assessment tools were used for different study designs. The risk of bias of included randomized controlled trials was evaluated using the Cochrane Collaboration Risk of Bias Tool. The quality of the evidence in the nonrandomized studies was assessed using Risk of Bias in Nonrandomized Studies of Interventions (ROBINS-I). The quality of review articles was assessed using the Oxman and Guyatt Criteria for a quality rating of systematic reviews. Qualitative studies were assessed using the critical appraisal checklist for qualitative research studies according to Treloar et al [9]. Each item in the assessment criteria was given a score of 1 if it was fulfilled (a negative item is considered fulfilled if it is avoided) in the article, and a score of 0 if was not fulfilled or if there was insufficient evidence to make a clear statement. Where a criterion was not considered by the study, it was marked as not applicable. The total score of each study was calculated by dividing the number of items included by the number of applicable items, yielding a score between 0 and 1. The methodological quality was considered low if the score was between 0 and 0.5 and high if the score was between 0.51 and 1.

### Synthesis of Results

Once data extraction was completed, the findings were grouped into themes based on their context. Data within the same theme were compared, and similarities and differences were identified between studies. A meta-analysis of the studies could not be carried out due to heterogeneity in the type of interventions and the nature of the research question, which focuses on user feedback.

## Results

### Study Selection

**Figure 1:**
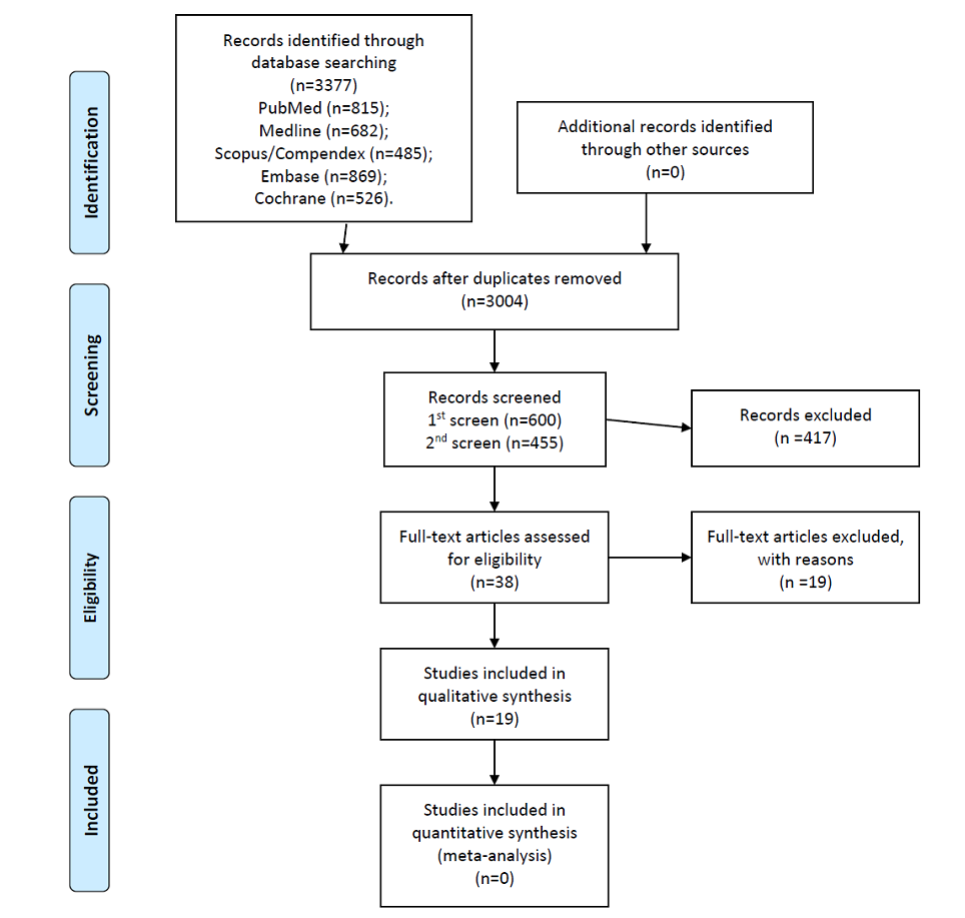
PRISMA (Preferred Reporting Items for Systematic Reviews and Meta-Analyses) study flow diagram.

The searches identified 3377 publications (Figure 1). After the removal of duplicates, 3004 relevant publications remained. Screening these results using keywords in the title and abstract led to 455 articles. The abstract of all 455 articles were read manually, which led to the identification of 38 potentially eligible publications. All 38 articles underwent full-text reading, which resulted in the inclusion of 19 studies [4,7,8,10-25]. The reasons for exclusion were the lack of an intervention (n=5), nontarget group of participants (not all overweight or obese; n=4), lack of a mention of specific features of wearable technology (n=4), lack of appropriate measurement of the experimental outcome (n=3), wrong type of wearable technology used during the intervention (n=1), and wearable technology used only as a means of intervention delivery (n=1, Multimedia Appendix 3).

### Study Characteristics

The study duration ranged from 3 weeks to 24 months [22], with 6 months being the most common intervention period [12,17,23]. The most common way to deliver the wearable device intervention was by a mobile app [11-15,17,20,23]. Ten studies collected postintervention user feedback by conducting interviews or group discussions [10-12,15,21].

All studies included some aspects of the social cognitive theory (Table 1). Self-determination [10-13], adaptive goals [10,13-15], and social support [10,12,14,18,22] were among the most frequently mentioned behavioral theories mentioned for designing the intervention. Some studies incorporated additional constructs, for example, the importance of cultural facilitators [11]. One study paired participants to give each other direct support and a sense of competition [14].

**Table 1:** Central motivation theory in included studies.

Central motivation theory identified in participants’ responses	Included study
Social support/competition	Donnachie et al (2017) [10] Lee and Kim (2016) [22] Mummah et al (2016) [12] Laing et al (2014) [18] Eisenhauer et al (2016) [19] Burke et al (2009) [23] Carter et al (2013) [24] Choo et al (2016) [14]
Self-determination	Donnachie et al (2017) [10] Maglalang et al (2017) [11] Robinson et al (2013) [16] Mummah et al (2016) [12] Naslund et al (2016) [13]
Adaptive goals	Donnachie et al (2017) [10] Hekler et al (2017) [15] Naslund et al (2016) [13] Martin et al (2015) [21] Burke et al (2009) [23] Choo et al (2016) [14]

### Methodological Quality

The methodological quality of the included studies was relatively weak, with 4 of 19 studies scoring below 0.5 (Tables 2-5). Overall, RCTs received the lowest score for methodological quality due to the general lack of allocation concealment and blinding. This is due to the nature of the wearable device intervention, which, in most cases, requires the researcher to give instructions to participants in the intervention group on how to use the device correctly. Similarly, participants must also understand the intervention they were given to abide by the details of the wearable technology intervention. However, two studies used blinding of investigator/assessor to increase study accuracy [18,21].

**Table 2:** Quality scores for randomized controlled trial calculated using the Cochrane Collaboration Risk of Bias Tool.

Study	Random sequence generation	Allocation concealment	Blinding	Incomplete outcome data	Selective reporting	Score
Carter et al (2013) [24]	1	0	0	0	1	0.40
Bentley et al (2016) [17]	1	0	0	0	1	0.40
Laing et al (2014) [18]	1	1	1	1	1	1.00
Martin et al (2015) [21]	1	0	1	1	1	0.80
Burke et al (2009) [23]	1	0	0	1	1	0.60

**Table 3:** Quality scores for nonrandomized studies (Risk of Bias in Nonrandomized Studies - of Interventions [ROBINS-I]).

Study	Confounding	Selection of participants	Classification of interventions	Deviations from intended interventions	Missing data	Outcome measurement	Selection of the reported result	Score
Choo et al (2016) [14]	1	0	1	1	0	0	0	0.43
Korinek et al (2017) [15]	1	1	1	1	0	0	1	0.71
Lee and Kim (2016) [22]	1	0	1	1	0	0	0	0.43
Naslund et al (2016) [13]	1	1	1	1	0	1	1	0.86
Eisenhauer et al (2016) [19]	1	0	1	1	1	1	1	0.86
Robinson et al (2013) [16]	1	0	1	1	1	1	1	0.86

**Table 4:** Quality scores of the qualitative studies (critical appraisal checklist for qualitative research studies).

Study	Purpose clear	Rationale appropriate	Conceptual framework	Ethical implications	Sampling strategy	Data collection procedures	Data organization	Data analysis	Reliability and validity in data collection and analysis	Progression from research question to conclusions	Score
Donnachie et al (2017) [10]	1	1	1	1	1	1	1	1	1	1	1.00
Huberty et al (2015) [20]	1	1	1	0	1	1	1	1	1	1	0.90
Maglalang et al (2017) [11]	1	1	1	1	0	1	1	1	1	1	0.90
Mummah et al (2016) [12]	1	1	1	1	1	1	0	1	1	1	0.90
Karduck et al (2018) [25]	1	1	0	1	0	1	1	1	1	1	0.80

**Table 5:** Review articles selected as per the Oxman and Guyatt criteria.

Study	Questions and method clearly stated	Comprehensive search methods	Inclusion explicit	Validity of primary studies	Assessment of the primary studies reproducible	Variation in the findings analyzed	Findings of the primary studies combined appropriately	Conclusions supported by the data cited	Overall risk
Bardus et al (2015) [7]	1	1	1	0	N/A^a^	1	1	1	0.86
Khaylis et al (2010) [8]	1	1	1	0	N/A	1	1	1	0.86
Lyzwinski et al (2014) [4]	1	1	1	1	1	1	1	1	1.00

^a^N/A: not applicable.

### Results of Individual Studies and Synthesis

Several themes were found, which are described below.

#### Self-efficacy

Participants found pedometers to be “a catalyst that enabled a new or renewed sense of self” [10]. Others expressed that a Fitbit accelerometer helped them “progress...from despair to self-efficacy” [11] and “improved participant’s self-efficacy in making healthy behavior changes” [11]. Participants in another study commented that they were “inspired” to increase their vegetable consumption [12]. Similarly, wearable devices have been “empowering” because they “helped create a sense of accomplishment from being more active and collecting more steps” [13]. On the other hand, a study found that participants who were more externally motivated failed to develop self-efficacy and were more likely to discontinue use of the device postintervention [10].

#### Goal Setting

Having a “goal” to work toward was identified by participants as a motivation to use the wearable. For example, participants commented that instantaneous feedback from the pedometer enabled them to “determine precisely how far they were from their goal” [10]. Some users perceived the device as a challenge: “It was like the one on one with myself and the Fitbit” [13]. In one study, “personal goal setting and monitoring” received the best feedback [14]. Variation in goals was also popular; one study showed that “100% of participants liked receiving different daily goals” [15]. However, some reported a “feeling of disappointment when unable to fulfil step targets” and discontinued use after the program [10].

#### Awareness

Participants commented that pedometers provided them with “an awareness of their (in)activity levels, which they felt they could not contest” [10]. Some felt that the mere presence of the app on their mobile phone and having to photograph and record foods “raised awareness of what they had been eating.” In addition, at times, this information resulted in changes in decision making regarding future eating [16]. Participants in other studies agreed that wearable mobile health devices “make you think and do things differently” [17] and are “helpful for increasing awareness of physical activity” [12]. Using a wearable “increases awareness of unhealthy food choices or portion size” [18] and “improved self-awareness about physical activity, water intake and portion sizes” [19]. Moreover, it provided an opportunity for the participants to “look at” their sleep quality (and confirm bad sleep quality) [20].

#### Feedback

Some participants felt that pedometer feedback gave them “personally relevant information and a meaningful rationale for increasing their activity levels” [10]. Others, while interested in receiving notification messages, “varied notably in their desired frequency” [12] of such notifications. “Encouraging messages” were appreciated by participants in a study, who felt that the messages “compelled (them)...to work towards their daily step goal” [13]. Similar results were also found in other studies, where participants “enjoyed receiving feedback on their progress,” “enjoyed the reminder feature” [18], “found specific suggestions helpful” [21], and “found the daily text messages...to be positive resources for self-monitoring eating and activity” [19]. In one study, participants identified the feedback they received from researchers as “their biggest motivator to wearing the sensor” [20]. Similarly, “feedback on physical activities” was one of the two features that had the highest satisfaction level [14].

#### Social Network and Communication

This construct received mixed feedback from users. Some participants found that the “interactional context” was one of the greatest sources of motivation; they felt that “connection to a group they valued” combined with a “perceived need/desire to report back to this group” kept them going [10]. The importance of meaningful connection was reflected in another study where researchers found that a more direct and closer relationship (eg, opposite sex, same occupation, and shared workspace) between the competing users resulted in more positive physical characteristic changes [22]. Users also “liked comparing their physical activity with other participants,” which “provides relevance to their self-monitoring” [19]. The two studies that received the most positive feedback on communication and competition were studies conducted in men only [10,19]. Most participants were interested in competing with others and “liked the way [the app] ranked everybody” [12]. However, some were “not interested in competing against friends and family outside of the study,” since “they might become discouraged if they were too far behind” [12]. Likewise, in two other studies, the overall use of the social networking feature was minimal, and satisfaction with the social networking service was low [14,18].

#### Acceptability in Social Settings

Participants were in favor of wearable devices and reported that the device was “socially acceptable.” They could “record in any public setting without having to let others know that they were self-monitoring” [23]. Smartphones gave them a “higher level of comfort using the study equipment in social settings” [24]. In some social situations, however, use of a mobile phone has been regarded as “inappropriate” and this decreased use [16].

#### Having Fun

In one study, participants commented that “self-monitoring with the pedometer provided an optimal challenge, which was fun/enjoyable in itself” [10]. Participants in other studies reported wanting “ideas” in the intervention that overcame the “boredom” of repeating the same activities repeatedly [12]. Similarly, in another study, participants who adhered to using wearable technology reported that it was “fun to use” [18], while the majority who gave up reported that it was “tedious” [18,19].

#### Suitability and Attractiveness of the Wearable Device

Comfort and appearance were among the primary considerations for using a device. Overall, participants found wearable technology “easy to use, portable and non-intrusive” [10]. Some commented that the device was “easy to incorporate into their everyday lives,” and, in some cases, linked it directly to adhering to increased exercise or dieting [17]. In addition, 30% of participants in one study even commented that they “would not have volunteered for the trial if there had been no offer of using a smartphone” [24]. In a study where three wearable devices were compared, the device that was the most comfortable received the highest satisfaction. In the same study, “appearance” was commonly referred to by female participants [20]. Some concerns about wearable technology included “frustration that it did not include more user-friendly software” [23], “desired better accuracy and precision in all aspects of the activity monitor” [19], and “challenges with...learning to use the Fitbit” [13].

## Discussion

### Principal Results

This review found 19 studies that reported user perspectives of wearable technology for weight management in people who are overweight and obese. It provides insight into what people have found to be helpful for their weight management when using a wearable device. Several themes were found, including “self-efficacy,” “goal setting,” “physical awareness,” “feedback,” “social comparison,” “acceptability in social settings,” “enjoyability,” and “attractiveness of hardware.” Participants had different views on “self-efficacy” and “goal setting,” indicating the importance of identifying and tailoring interventions to individual motivations in order to facilitate technology adoption. Furthermore, specific design elements were identified by users as decreasing their likelihood of adopting the technology. For example, while a smartphone was considered an attractive means of intervention delivery, users sometimes found it inappropriate in social settings. Users also found some types of devices, such as waist-worn ones, not comfortable enough to be worn all the time. Feedback from the device indicating that it had been worn properly was also appreciated by users.

### Limitations

The methodological quality of the included studies was relatively weak, with 4 of 19 studies scoring below 0.5. Most studies had a relatively short duration, with an average of 6 months, which means that they could not provide evidence for the long-term effectiveness of wearable technology. Long-term adoption of technology and sustained weight loss are challenging. Only a few studies conducted a thorough qualitative analysis of the participants’ views to help identify essential features of the intervention that made it a successful weight loss intervention. A carefully charted tool for obtaining comprehensive user feedback may be the first step to achieving this.

A strength of this review is that we followed, where possible, the comprehensive Cochrane Collaboration methods for systematic reviews. A limitation of this review is that we did not hand search journals or the grey literature. However, a comprehensive search of electronic databases was conducted to find relevant studies.

### Implications for Policy and Practice

The effect of different theories varies when approaching different groups of individuals, including hard-to-reach populations. For example, cultural factors play a significant role in treating at-risk ethnic minorities, and special adaptations may be required in treating patients with a mental illness. In addition, interventions targeting rural populations may require special attention to access disparity and seasonal exercise level fluctuation. Sources of motivation also play a part in achieving optimal results, and intervention programs need to be tailored to individuals with different needs. Hence, it may be worth considering introducing a preintervention assessment to make sure participants are introduced to an intervention that suits their individual needs.

### Future Research

For future studies to provide more meaningful information on user feedback, the criteria used by researchers in qualitative data collection should be specified. Participants might then be guided to provide a more in-depth and comprehensive reflection on their intervention experience and on the features they felt were helpful or needed. This information is much needed for the future development of wearable technology for weight loss to meet the specific needs of people who are overweight or obese. Comfort and appearance were important considerations for using a device. Thus, further research should develop wearable devices that are comfortable to wear and acceptable in social settings, for example, digital glasses.

### Conclusions

Our review found mixed views on the effective use of wearable technology for weight loss interventions. This highlights the importance of determining the type of intervention most suitable for an individual before the intervention is used.

## Appendix

Multimedia Appendix 1 PRISMA (Preferred Reporting Items for Systematic Reviews and Meta-Analyses) checklist.

Multimedia Appendix 2 Full search strategies used.

Multimedia Appendix 3 Eligibility stage exclusions.

## Abbreviations

PICO: Participants, Interventions, Comparators, Outcomes
PRISMA: Preferred Reporting Items for Systematic Reviews and Meta-Analyses
ROBINS-I: Risk of Bias in Nonrandomized Studies - of Interventions
