# Supplementary material for: Perspectives of People Who Are Overweight and Obese on Using Wearable Technology for Weight Management: Systematic Review
Source: JMIR Mhealth Uhealth. 2020 Jan 13;8(1):e12651. doi: 10.2196/12651 (PMC6996738; doi:10.2196/12651)
Supplement: Multimedia Appendix 2 [file mhealth_v8i1e12651_app2.docx]

Appendix B: Full search strategies used

| Database | MeSH Terms | Keywords |
| --- | --- | --- |
| Pubmed | (Adult[MeSH Terms]) AND (equipment design[MeSH Terms] OR patient satisfaction[MeSH Terms] OR attitude[MeSH Terms] OR qualitative research[MeSH Terms] OR user-computer interface[MeSH Terms] OR software[MeSH Terms]) AND (Weight Loss[MeSH Terms] OR Weight Gain[MeSH Terms] OR Body Weight[MeSH Terms] OR Body Mass Index[MeSH Terms] OR Adipose Tissue[MeSH Terms] OR adipocytes[MeSH Terms] OR Subcutaneous Fat, Abdominal[MeSH Terms] OR Adipocytes, White[MeSH Terms] OR Body Composition[MeSH Terms] OR Body Size[MeSH Terms] OR Body Weights[MeSH Terms] AND Measures[MeSH Terms] OR Subcutaneous Fat[MeSH Terms] OR Abdominal Fat[MeSH Terms] OR Body Weight Maintenance[MeSH Terms] OR Body Weight Changes[MeSH Terms] OR Adipose Tissue, White[MeSH Terms]) AND (Wearable Electronic Devices[MeSH Terms] OR physical activity tracker[MeSH Terms] OR accelerometry[MeSH Terms] OR smartphone[MeSH Terms] OR wearable device[MeSH Terms] OR mobile applications[MeSH Terms]) AND (obesity, metabolically benign[MeSH Terms] OR obesity[MeSH Terms] OR obesity, abdominal[MeSH Terms] OR obesity, morbid[MeSH Terms] OR overweight[MeSH Terms]) AND (“last 10 years”[PDat]) | (Adult[MeSH Terms]) AND ("equipment design*" OR "interface design*" OR "software design*" OR "user* feedback" OR "user satisfaction" OR "consumer* feedback" OR "consumer* satisfaction" OR "consumer* experience" OR "user* experience*" OR "user opinion*" OR "user rating*" OR "patient* opinion*" OR "user* suggestion*" OR "user review*" OR "user* interface*" OR "user-computer interface*" OR "product* design" OR "design feature*" OR "unique feature*" OR "evaluation form*" OR "subjective result*" OR "subjective experimental result*" OR “appreciation*") AND ("weight loss" OR "weight gain" OR weightloss OR "weight change" OR "weight reduction" OR "weight management" OR "body mass index" OR bmi OR adipo* OR "body composition*" OR "body size*" OR "weight measur*" OR "subcutaneous fat*" OR "abdominal fat*" OR "long term" OR long-term OR "long lasting" OR "long-lasting" OR adher* OR compliance* OR comply) AND (“fitness track*" OR "activity track" OR "activity monitor*" OR "movement track*" OR "movement sens*" OR "movement monitor*" OR "self monitoring device*" OR acceleromet* OR "step count*" OR inclinomet* OR "self track*" OR fitbit* OR "wearable technolog*" OR "wearable device*" OR "fitness sens*" OR pedomet* OR "mobile application*" OR "electronic wristband*" OR "electronic monitoring device*" OR "smart watch*" OR "electronic wristband*" OR "electronic bracelet*" OR "smart phone application*" OR "mobile app*" OR "smartphone app*”) AND (obes* OR overweight* OR over weight* OR BMI>25 OR BMI>30)) OR (obes* OR overweight* OR "over weight*" OR BMI>25 OR BMI>30) AND "last 10 years"[PDat] |
| Embase  (Ovid) | (equipment design/ or patient satisfaction/ or user-computer interface/ or software/ or attitude/ or qualitative research/) AND (Wearable Electronic Devices/ or physical activity tracker/ or accelerometry/ or smartphone/ or wearable device/ or mobile applications/) AND (Weight Loss/ or Weight Gain/ or Body Weight/ or Body Mass Index/ or Adipose Tissue/ or adipocytes/ or Subcutaneous Fat, Abdominal/ or Adipocytes, White/ or Body Composition/ or Body Size/ or Subcutaneous Fat/ or Abdominal Fat/ or Body Weight Maintenance/ or Body Weight Changes/ or Adipose Tissue, White/) AND (obesity, metabolically benign/ or obesity/ or obesity, abdominal/ or obesity, morbid/ or overweight/)  Limit to (english language and yr="2008 - 2018" and "all adult (19 plus years)”) | (equipment design* or interface design* or software design* or user* feedback or user satisfaction or consumer* feedback or consumer* satisfaction or consumer* experience or user* experience* or user opinion* or user rating* or patient* opinion* or user* suggestion* or user review* or user* interface* or user-computer interface* or product* design or design feature* or product feature* or unique feature* or desired feature* or popular feature* or evaluation form* or subjective result* or subjective experimental result* or appreciation* or qualitative research) AND ((fitness track* or activity track* or activity monitor* or movement track* or movement sens* or movement monitor* or self-monitoring device* or acceleromet* or step count* or inclinomet* or self-track* or fitbit* or wearable technolog* or wearable device* or fitness sens* or pedomet* or mobile application* or electronic wristband* or electronic monitoring device* or smart watch* or electronic wristband* or electronic bracelet* or smart phone application* or mobile app* or smartphone app*) AND (weight loss or weight gain or weightloss or weight change or weight reduction or weight management or body mass index or bmi or adipo* or body composition* or body size* or weight measur* or subcutaneous fat* or abdominal fat* or long-term or long term or long lasting or long-lasting or adher* or compliance or comply) AND (obes* or overweight* or over weight* or BMI 25 or BMI 30)  Limit to (english language and yr="2008 - 2018" and "all adult (19 plus years)”) |
| Cochrane Library | (([Wearable Electronic Devices] or [Fitness Trackers] or [Smartphone] or [Mobile Applications])explode all trees) and (([Weight Loss] or [Weight Gain] or [Body Mass Index] or [Adipose Tissue] or [Subcutaneous Fat]) explode all trees) and ([Obesity] or [overweight])  (No MeSH terms for user experience - use keywords instead) | (fitness track* or activity track* or activity monitor* or movement track* or movement sens* or movement monitor*or self monitoring device* or acceleromet* or step count* or inclinomet* or self track* or fitbit* or wearable technolog* or wearable device* or fitness sens* or pedomet* or mobile application* or smartphone application* or smart phone application* or mobile application* or mobile app* or smartphone app* or smartphone* or wearable electronic device* or accelerometr*:ti,ab,kw) and (weight loss or weight gain or weightloss or weight change or weight reduction or weight management or body mass index or bmi or adipo* or body composition* or body size* or weight measur* or subcutaneous fat* or abdominal fat* or long term or long-term or long lasting or long-lasting or adher* or compliance* or comply:ti,ab,kw) and (Survey* or questionnaire* or equipment design* or interface design* or software design* or user* feedback or user satisfaction or consumer* feedback or consumer* satisfaction or consumer* experience or user* experience* OR user opinion* OR user rating* OR patient* opinion* OR user* suggestion* OR user review* OR user* interface* OR user-computer interface* OR product* design OR design feature* OR unique feature* OR evaluation form* OR subjective result* OR subjective experimental result* OR appreciation* OR qualitative study:ti,ab,kw) and (obes* or overweight* or over weight*:ti,ab,kw) |
| Scopus/  Compendex | N/A | (Adult[MeSH Terms]) AND ("equipment design*" OR "interface design*" OR "software design*" OR "user* feedback" OR "user satisfaction" OR "consumer* feedback" OR "consumer* satisfaction" OR "consumer* experience" OR "user* experience*" OR "user opinion*" OR "user rating*" OR "patient* opinion*" OR "user* suggestion*" OR "user review*" OR "user* interface*" OR "user-computer interface*" OR "product* design" OR "design feature*" OR "unique feature*" OR "evaluation form*" OR "subjective result*" OR "subjective experimental result*" OR “appreciation*") AND ("weight loss" OR "weight gain" OR weightloss OR "weight change" OR "weight reduction" OR "weight management" OR "body mass index" OR bmi OR adipo* OR "body composition*" OR "body size*" OR "weight measur*" OR "subcutaneous fat*" OR "abdominal fat*" OR "long term" OR long-term OR "long lasting" OR "long-lasting" OR adher* OR compliance* OR comply) AND (“fitness track*" OR "activity track" OR "activity monitor*" OR "movement track*" OR "movement sens*" OR "movement monitor*" OR "self monitoring device*" OR acceleromet* OR "step count*" OR inclinomet* OR "self track*" OR fitbit* OR "wearable technolog*" OR "wearable device*" OR "fitness sens*" OR pedomet* OR "mobile application*" OR "electronic wristband*" OR "electronic monitoring device*" OR "smart watch*" OR "electronic wristband*" OR "electronic bracelet*" OR "smart phone application*" OR "mobile app*" OR "smartphone app*”) AND (obes* OR overweight* OR over weight* OR BMI>25 OR BMI>30)) OR (obes* OR overweight* OR "over weight*" OR BMI>25 OR BMI>30) AND PUBYEAR AFT 2008 |
