# Supplementary material for: Perspectives of People Who Are Overweight and Obese on Using Wearable Technology for Weight Management: Systematic Review
Source: JMIR Mhealth Uhealth. 2020 Jan 13;8(1):e12651. doi: 10.2196/12651 (PMC6996738; doi:10.2196/12651)
Supplement: Multimedia Appendix 3 [file mhealth_v8i1e12651_app3.doc]

### Appendix C: Eligibility Stage Exclusions

**Manually excluded after abstract review**

| **Study title** | **Reason for exclusion** |
| --- | --- |
| Differences in fat loss in response to physical activity among severely obese men and women | Wearable technology used only as a means of measurement |
| Techniques to measure free-living energy expenditure during pregnancy - A guide for clinicians and researchers | Wearable technology used only as a means of measurement |
| Comparison of different methods: Used in evaluation of physical activity | Wearable technology used only as a means of measurement |
| Improvements in weight, HbA1C and fitness following lifestyle intervention: The PULSE trial for type 2 diabetes prevention in men | No emphasis on specific features of wearable technology |
| The life styles causing overweight or obesity: Based on 5 years of experience in two centers in Sulaimani Governorate, Kurdistan Region/Iraq | No wearable intervention |
| Impact of an mHealth-supported behavioural lifestyle intervention on exercise stage-of-change and physical activity in overweight and obese pregnancy: PEARs randomised controlled trial (RCT) | Focus was on PA and not weight loss |
| Impact of a smartphone app supporting a lifestyle intervention in overweight and obese pregnancy on on maternal health and lifestyle outcomes | No weight loss outcome data |
| Impact of an mhealth-supported behavioral lifestyle intervention on behavioral stage of change and physical activity in overweight and obese pregnancy: Pears randomized controlled trial | Duplicate |
| Effectiveness of health and fitness smartphone applications to improve dietary habits and physical activity in Omani adults | Study not carried out on overweight and obese individuals only |
| Technology-Assisted Weight Management Interventions: Systematic Review of Clinical Trials | Includes Web-based studies |
| Comparing accelerometer and international physical activity questionnaire for assessing physical activity levels | Accuracy assessment of wearable technology |
| Feasibility of a commercial smartphone application for dietary assessment in epidemiological research and comparison with 24-h dietary recalls | Study not carried out on overweight and obese individuals only |
| Feasibility study to assess the impact of a lifestyle intervention (a LivingWELL') in people having an assessment of their family history of colorectal or breast cancer | Wearable technology used only as a means of measurement |
| Feasibility study to assess the impact of a lifestyle intervention ('LivingWELL') in people having an assessment of their family history of colorectal or breast cancer | Duplicate |
| Social cognitive determinants of nutrition and physical activity among web-health users enrolling in an online intervention: the influence of social support, self-efficacy, outcome expectations, and self-regulation | Demographic study |
| Use of a computerized tracking system to monitor and provide feedback on dietary goals for calorie-restricted diets: The POUNDS LOST study | Wearable technology used only as a means of measurement |
| Use of a computerized tracking system to monitor and provide feedback on dietary goals for calorie-restricted diets: the POUNDS LOST study | Duplicate |
| Use of a Computerized Tracking System to Monitor and Provide Feedback on Dietary Goals for Calorie-Restricted Diets: The POUNDS LOST Study | Duplicate |
| Validation of a novel protocol for calculating estimated energy requirements and average daily physical activity ratio for the US population: 2005-2006. | Not a weight loss intervention |
| Cadence Feedback With ECE PEDO to Monitor Physical Activity Intensity: A Pilot Study | Study not carried out on overweight and obese individuals only |
| Cadence feedback with ECE PEDO to monitor physical activity intensity: A pilot study | Duplicate |
| Cadence Feedback With ECE PEDO to Monitor Physical Activity Intensity: A Pilot Study | Duplicate |
| Endometrial cancer survivors' sleep patterns before and after a physical activity intervention: A retrospective cohort analysis | Not a weight loss intervention |
| Feasibility of Behavioral Weight Loss Treatment Enhanced with Peer Support and Mobile Health Technology for Individuals with Serious Mental Illness | Wearable technology isn’t the focus of the intervention |
| Feasibility of Behavioral Weight Loss Treatment Enhanced with Peer Support and Mobile Health Technology for Individuals with Serious Mental Illness | Duplicate |
| Associations of moderate-to-vigorous-intensity physical activity and body mass index with glycated haemoglobin within the general population: a cross-sectional analysis of the 2008 Health Survey for England | Wearable technology used only as a means of measurement |
| Associations of moderate-to-vigorous-intensity physical activity and body mass index with glycated haemoglobin within the general population: a cross-sectional analysis of the 2008 Health Survey for England | Duplicate |
| Development and implementation of a church-based eHealth program to reduce obesity in Appalachian adults | Web-based intervention |
| Comparing physical activity measures in a diverse group of midlife and older adults | Wearable technology used only as a means of measurement |
| A systematic review of motivational interviewing for weight loss among adults in primary care | Wearable technology isn’t the focus of the intervention |
| myPace: an integrative health platform for supporting weight loss and maintenance behaviors | Duplicate |
| “Free-living" standing, sitting and lying times among sedentary adults in different BMI categories | Demographic study |
| ActiGraph GT3X determined variations in "free-living" standing, lying and sitting duration among sedentary adults | Demographic study |
| Health4families: A behavioral intervention to improve weight and health behaviors in Lynch syndrome families | Wearable technology isn’t the focus of the intervention |
| Tailored physical activity interventions as innovative means to address women's perinatal mental health | Study not carried out on overweight and obese individuals only |
| Assessment tools in obesity - Psychological measures, diet, activity, and body composition | Accuracy assessment of wearable technology |
| Smartphone application for real-time dietary assessment and physical activity analyses in dietary counseling | Face-to-face counseling |
| Healthy obesity and objective physical activity | Wearable technology used only as a means of measurement |
| Measuring physical activity in pregnancy: a comparison of accelerometry and self-completion questionnaires in overweight and obese women | Wearable technology used only as a means of measurement |
| Reduced physical activity in stable renal transplanted patients | Wearable technology used only as a means of measurement |
| Lifestyle and dietary habits in renal transplanted patients | Wearable technology used only as a means of measurement |
| An in situ study of the habits of users that affect office chair design and testing | Wearable technology isn’t the focus of the intervention |
| Feasibility study of portable technology for weight loss and HbA1c control in type 2 diabetes | Duplicate |
| Increasing physical activity in office workers--the Inphact Treadmill study; a study protocol for a 13-month randomized controlled trial of treadmill workstations | Wearable technology used only as a means of measurement |
| Action control in dyads: A randomized controlled trial to promote physical activity in everyday life | Focus was on PA and not weight loss |
| Physical Activity, BMI, and Blood Pressure in US Youth: NHANES 2003-2006 | Demographic study |
| A randomised controlled trial of a physical activity and nutrition program targeting middle-aged adults at risk of metabolic syndrome in a disadvantaged rural community | Focus was on PA and not weight loss |
| Impact of self-help weight loss resources with or without online support on the dietary intake of overweight and obese men: the SHED-IT randomised controlled trial | Web-based intervention |
| Adults with parental history of type 2 diabetes who curtail their sleep have reduced physical activity | Focus was on PA and not weight loss |
| Association between objectively measured physical activity and mortality risk in adults | Focus was on PA and not weight loss |
| Determinants of overweight and obesity in lung transplant recipients | Wearable technology used only as a means of measurement |
| Determinants of overweight and obesity in lung transplant recipients | Duplicate |
| Text Messaging as Adjunct to Community-Based Weight Management Program | Text messaging |
| Neighborhood design for walking and biking: physical activity and body mass index | Wearable technology used only as a means of measurement |
| Using accelerometer feedback to identify walking destinations, activity overestimates, and stealth exercise in obese and nonobese individuals | Wearable technology used only as a means of measurement |
| Training overweight/obese older women at high risk for breast cancer to use web-based weight loss tools: The HELP pilot study | Web-based intervention |
| Training overweight/obese older women at high risk for breast cancer to use web-based weight loss tools: The HELP pilot study | Duplicate |
| Behavioral risk factors of chronic non-communicable diseases in medical doctors | Wearable technology used only as a means of measurement |
| Digital food guide: Mobile application to incorporate healthy food choices | Study not carried out on overweight and obese individuals only |
| Physical activity, body fatness, and visceral adiposity in overweight latino and non-latino adults | Wearable technology used only as a means of measurement |
| Physical activity and sedentary behavior in metabolically healthy obese young women | Focus was on PA and not weight loss |
| Lifestyle Behaviors in Metabolically Healthy and Unhealthy Overweight and Obese Women: A Preliminary Study | Demographic study |
| Maintaining physical activity during an energy-restricted diet is as beneficial for insulin sensitivity as weight loss | Wearable technology used only as a means of measurement |
| Measuring physical activity level, and sedentary behaviours among students at the university of medicine, Albania | Focus was on PA and not weight loss |
| Weight Bias and Weight Loss Treatment Outcomes in Treatment-Seeking Adults | Wearable technology isn’t the focus of the intervention |
| Brief physical activity-related psychosocial measures: reliability and construct validity | Focus was on PA and not weight loss |
| PREventive Care Infrastructure based On Ubiquitous Sensing (PRECIOUS): A Study Protocol | Protocol |
| Correlates of overall and central obesity in adults from seven European countries: findings from the Food4Me Study | Demographic study |
| Subjective and objective appraisal of activity in adults with obstructive sleep apnea | Wearable technology used only as a means of measurement |
| Insomnia and physical activity in adults with prediabetes | Wearable technology used only as a means of measurement |
| Insomnia and physical activity in adults with prediabetes | Duplicate |
| Effect of insulin resistance improvement due to lifestyle intervention on overweight perimenopausal Japanese women: a preliminary study | Wearable technology isn’t the focus of the intervention |
| Physical activity and physical function in older adults with knee osteoarthritis | Focus was on PA and not weight loss |
| Physical activity and physical function in older adults with knee osteoarthritis | Duplicate |
| Exercise amount calculation using a wearable half-cell potential sensor for mobile aerobic exercise management | Accuracy assessment of wearable technology |
| Exercise amount calculation using a wearable half-cell potential sensor for mobile aerobic exercise management | Duplicate |
| More than telemonitoring: Health provider use and nonuse of life-log data in irritable bowel syndrome and weight management | Study not carried out on overweight and obese individuals only |
| More than telemonitoring: Health provider use and nonuse of life-log data in irritable bowel syndrome and weight management | Duplicate |
| Enhanced insulin sensitivity in successful, long-term weight loss maintainers compared with matched controls with no weight loss history | Wearable technology isn’t the focus of the intervention |
| Evaluation of a commercially available pedometer used to promote physical activity as part of a national programme | Study not carried out on overweight and obese individuals only |
| Limiting excess weight gain in healthy pregnant women: importance of energy intakes, physical activity, and adherence to gestational weight gain guidelines | Wearable technology used only as a means of measurement |
| The association between discrepant weight perceptions and objectively measured physical activity | Wearable technology used only as a means of measurement |
| Reallocating time between sleep, sedentary and active behaviours: Associations with obesity and health in Canadian adults | Demographic study |
| Habitual physical activity in pre-, peri- and postmenopause: Anthropometrics and cardiovascular risk factors in a cohort of women in southern Brazil | Wearable technology used only as a means of measurement |
| Step counts and sedentary time in type 2 diabetes and hypertension: Seasonal variations | Wearable technology used only as a means of measurement |
| Characterizing vascular health in female nurses in the champlain region of Ontario | Demographic study |
| The Alberta moving beyond breast cancer (AMBER) cohort study: Recruitment, baseline assessment, and description of the first 500 participants | Wearable technology used only as a means of measurement |
| Predictors of physical activity and fitness in a 6-month, home-based physical activity program for older adults: MOVES | Focus was on PA and not weight loss |
| Demographic and clinical correlates ofaccelerometer assessed physical activity and sedentary time in lung cancer survivors | Demographic study |
| Small-changes obesity treatment among veterans: 12-Month outcomes | Wearable technology used only as a means of measurement |
| Relationship between volition, physical activity and weight loss maintenance: Study rationale, design, methods and baseline characteristics | Wearable technology isn’t the focus of the intervention |
| The WATCH (Weight Activity and Time Contributes to Health) paradigm and quality of life: the impact of overweight/obesity duration on the association between physical activity and health-related quality of life | Wearable technology isn’t the focus of the intervention |
| Mild Depressive Symptoms Among Americans in Relation to Physical Activity, Current Overweight/Obesity, and Self-Reported History of Overweight/Obesity | Wearable technology isn’t the focus of the intervention |
| The Impact of Overweight/Obesity Duration and Physical Activity on Medical Multimorbidity: Examining the WATCH Paradigm | Wearable technology isn’t the focus of the intervention |
| Step Monitoring to improve ARTERial health (SMARTER) through step count prescription in type 2 diabetes and hypertension: Trial design and methods | No intervention carried out |
| Voluntary and controlled weight loss can reduce symptoms and allow a lowering of proton pump inhibitor dosage in patients with gastroesophageal reflux disease. A case-control study | Wearable technology isn’t the focus of the intervention |
| The evaluation of the efficacy of weight loss in controlling symptoms in patients with gastroesophageal reflux symptoms | Wearable technology used only as a means of measurement |
| The Netherlands Epidemiology of Obesity (NEO) study: study design and data collection | Study not carried out on overweight and obese individuals only |
| The Netherlands epidemiology of obesity (NEO) study: Study design and data collection | Duplicate |
| Long-term effects on physical activity and body weight after participation in a randomized weight loss trial in postmenopausal women | Study not carried out on overweight and obese individuals only |
| Long-term effects on physical activity and body weight after participation in a randomized weight loss trial in postmenopausal women | Duplicate |
| Men on the Move-Nashville: Feasibility and Acceptability of a Technology-Enhanced Physical Activity Pilot Intervention for Overweight and Obese Middle and Older Age African American Men | Text messaging |
| Prehabilitation for patients with end-stage liver disease | Wearable technology used only as a means of measurement |
| Preventing body fat gain in women being treated for breast cancer | Wearable technology used only as a means of measurement |
| It’s like a personal motivator that you carried around wi’ you’: utilising self-determination theory to understand men’s experiences of using pedometers to increase physical activity in a weight management programme | Duplicate |
| Weight management for individuals with intellectual and developmental disabilities: rationale and design for an 18 month randomized trial | No intervention carried out |
| Community resource utilization, psychosocial health, and sociodemographic factors associated with diet and physical activity among low-income obese Latino immigrants | Wearable technology used only as a means of measurement |
| Momentary assessment of adults' physical activity and sedentary behavior: Feasibility and validity | Wearable technology used only as a means of measurement |
| The impact of targeted exercise intervention on health outcomes in rheumatoid arthritis | Wearable technology used only as a means of measurement |
| Effects of the menopausal transition on factors related to energy balance in women with varying adiposity. a MONET study | Wearable technology used only as a means of measurement |
| Physical activity reduce hepatic apoptosis in patients with non-alcoholic fatty liver disease and visceral obesity | Wearable technology used only as a means of measurement |
| Chronic Health Risks in Commercial Fishermen: A Cross-Sectional Analysis from a Small Rural Fishing Village in Alaska | Wearable technology used only as a means of measurement |
| Irregular meals are associated with a higher body mass index in people with type 2 diabetes | Wearable technology used only as a means of measurement |
| Overweight prevalence and trends in Hungarian adults: The Hungarian diet and nutritional status surveys 2009 and 2014 | Demographic study |
| The NULevel trial of a scalable, technology-assisted weight loss maintenance intervention for obese adults after clinically significant weight loss: study protocol for a randomised controlled trial | Protocol |
| Changes in physical activity among postpartum overweight and obese women: results from the KAN-DO Study | Focus was on PA and not weight loss |
| Changes in physical activity among postpartum overweight and obese women: results from the KAN-DO Study | Duplicate |
| E-technology and pedometer walking program to increase physical activity at work | Study not carried out on overweight and obese individuals only |
| Moderate to vigorous physical activity and weight outcomes: does every minute count? | Wearable technology used only as a means of measurement |
| Pain worsening with physical activity during migraine attacks in women with overweight/obesity: A prospective evaluation of frequency, consistency, and correlates | Wearable technology used only as a means of measurement |
| Effects of body mass index and tilt angle on output of two wearable activity monitors | Accuracy assessment of wearable technology |
| Moderate-vigorous physical activity across body mass index in females: moderating effect of endocannabinoids and temperament | Wearable technology used only as a means of measurement |
| The effect of exercise consultation and exercise therapy on quality of life in women with type 2 diabetes: A randomized clinical trial | Wearable technology used only as a means of measurement |
| Eating control and physical activity as determinants of short-term weight maintenance after a very-low-calorie diet among obese women | Wearable technology used only as a means of measurement |
| PREVIEW: Prevention of diabetes through lifestyle intervention and population studies in Europe and around the world. design, methods, and baseline participant description of an adult cohort enrolled into a three-year randomised clinical trial | Results not yet generated |
| PREVIEW: Prevention of diabetes through lifestyle intervention and population studies in Europe and around the world. design, methods, and baseline participant description of an adult cohort enrolled into a three-year randomised clinical trial | Duplicate |
| Weight gain prevention among black women in the rural community health center setting: the Shape Program | Wearable technology isn’t the focus of the intervention |
| A randomized controlled trial of a digital health obesity treatment intervention for medically vulnerable primary care patients | Wearable technology isn’t the focus of the intervention |
| A randomized controlled trial of a digital health obesity treatment intervention for medically vulnerable primary care patients | Duplicate |
| Personalized Weight Management Interventions for Cardiovascular Risk Reduction: A Viable Option for African-American Women | Web-based intervention |
| The role of exercise training in a weight loss program on psychosocial morbidity, sleep quality and physical activity in obese asthmatics: A RCT | Wearable technology isn’t the focus of the intervention |
| A weight loss intervention using a commercial mobile application in latino americans-adelgaza trial | No emphasis on specific features of wearable technology |
| Association of physiological and psychological health outcomes with physical activity and sedentary behavior in adults with type 2 diabetes | Wearable technology used only as a means of measurement |
| Personalized Health, eLearning, and mHealth Interventions to Improve Nutritional Status | Web-based intervention |
| Diets, body composition, physical activity and fitness of first nations youth from kashechewan, ontario: An opportunity for collaborative initiatives to promote healthy weights | Demographic study |
| Promoting weight loss through diet and exercise in overweight or obese breast cancer survivors (InForma): study protocol for a randomized controlled trial | Protocol |
| Using social and mobile tools for weight loss in overweight and obese young adults (Project SMART): a 2 year, parallel-group, randomised, controlled trial | Duplicate |
| Application of Machine Learning to Predict Dietary Lapses During Weight Loss | Wearable technology isn’t the focus of the intervention |
| Physical Activity as a Vital Sign: A Systematic Review | Focus was on PA and not weight loss |
| Urban Planning and Health Inequities: Looking in a Small-Scale in a City of Cape Verde | Demographic study |
| Policy options for obesity in Europe: a comparison of public health specialists with other stakeholders | Demographic study |
| Health-specific information and communication technology use and its relationship to chronic disease status in communities on the South side of Chicago | Demographic study |
| STEPWISE - STructured lifestyle Education for People WIth SchizophrEnia: a study protocol for a randomised controlled trial | Protocol |
| STEPWISE - STructured lifestyle Education for People WIth SchizophrEnia: a study protocol for a randomised controlled trial | Duplicate |
| Feasibility and acceptance of exercise recommendations (10,000 steps a day) within routine German health check (Check-Up 35/GOA29)-study protocol | Protocol |
| Ascertaining the Place of Social Media and Technology for Bariatric Patient Support: What Do Allied Health Practitioners Think? | No emphasis on specific features of wearable technology |
| Can professional soccer clubs help male fans lose weight and become more physically active? Preliminary evidence from the Scottish Premier League | Wearable technology isn’t the focus of the intervention |
| Football Fans in Training: the development and optimization of an intervention delivered through professional sports clubs to help men lose weight, become more active and adopt healthier eating habits | Wearable technology isn’t the focus of the intervention |
| Sedentary behavior and food cravings in diverse overweight women: a pilot study | Wearable technology used only as a means of measurement |
| The effect of aerobic exercise on metabolic and inflammatory markers in breast cancer survivors--a pilot study | Wearable technology isn’t the focus of the intervention |
| The effect of aerobic exercise on metabolic and inflammatory markers in breast cancer survivors--a pilot study | Duplicate |
| Factors influencing telomere length in BRCA-mutation carriers | Wearable technology isn’t the focus of the intervention |
| Associations between lower extremity muscle mass and metabolic parameters related to obesity in Japanese obese patients with type 2 diabetes | Wearable technology used only as a means of measurement |
| Relationship between sleep and cardiometabolic factors in young adults | Wearable technology used only as a means of measurement |
| Evaluation of dietitian of canada (DC)'s eatracker mobile app | Study not carried out on overweight and obese individuals only |
| Patterns of objectively measured physical activity in normal weight, overweight, and obese individuals (20-85 years): a cross-sectional study | Wearable technology used only as a means of measurement |
| What factors are associated with physical activity in older people, assessed objectively by accelerometry? | Wearable technology used only as a means of measurement |
| Optimizing healthy gestational weight gain in women at high risk of gestational diabetes: a randomized controlled trial | Study not carried out on overweight and obese individuals only |
| Optimizing healthy gestational weight gain in women at high risk of gestational diabetes: a randomized controlled trial | Duplicate |
| Understanding early pregnancy health behaviours among overweight and obese women at risk of gestational diabetes mellitus | Wearable technology used only as a means of measurement |
| Limiting postpartum weight retention through early antenatal intervention: the HeLP-her randomised controlled trial | Wearable technology used only as a means of measurement |
| Association between objectively measured physical activity and body mass index with low back pain: a large-scale cross-sectional study of Japanese men | Wearable technology used only as a means of measurement |
| TXT2BFiT’ a mobile phone-based healthy lifestyle program for preventing unhealthy weight gain in young adults: study protocol for a randomized controlled trial | Text messaging |
| Tailored behavioral medicine intervention for enhanced physical activity and healthy eating in patients with obstructive sleep apnea syndrome and overweight | Wearable technology isn’t the focus of the intervention |
| Light physical activity determined by a motion sensor decreases insulin resistance, improves lipid homeostasis and reduces visceral fat in high-risk subjects: PreDiabEx study RCT | Wearable technology used only as a means of measurement |
| Food-related behavior, physical activity, and dietary intake in First Nations - a population at high risk for diabetes | Wearable technology used only as a means of measurement |
| Food-related behavior, physical activity, and dietary intake in First Nations - a population at high risk for diabetes | Duplicate |
| Compliance to step count and vegetable serve recommendations mediates weight gain prevention in mid-age, premenopausal women. Findings of the 40-Something RCT | Study not carried out on overweight and obese individuals only |
| Stages of change for physical activity and dietary habits in persons with type 2 diabetes included in a mobile health intervention: the Norwegian study in RENEWING HEALTH | Study not carried out on overweight and obese individuals only |
| abiliti Closed-Loop Gastric Electrical Stimulation System for Treatment of Obesity: Clinical Results with a 27-Month Follow-Up | Wearable technology isn’t the focus of the intervention |
| abiliti Closed-Loop Gastric Electrical Stimulation System for Treatment of Obesity: Clinical Results with a 27-Month Follow-Up | Duplicate |
| An Internet-Based Virtual Coach to Promote Physical Activity Adherence in Overweight Adults: Randomized Controlled Trial | Web-based intervention |
| Physical activity behaviour and sedentary time in persons with obstructive sleep apnea and obesity | Wearable technology used only as a means of measurement |
| Using open source accelerometer analysis to assess physical activity and sedentary behaviour in overweight and obese adults | Wearable technology used only as a means of measurement |
| Physical activity-related energy expenditure with the RT3 and TriTrac accelerometers in overweight adults | Accuracy assessment of wearable technology |
| Effect of wearable technology combined with a lifestyle intervention on long-term weight loss: The IDEA randomized clinical trial | No user experience component |
| Effect of wearable technology combined with a lifestyle intervention on long-term weight loss: The IDEA randomized clinical trial | Duplicate |
| Accuracy of self-reported exercise and the relationship with weight loss in overweight women | Wearable technology used only as a means of measurement |
| Smartphone Usage, Social Media Engagement, and Willingness to Participate in mHealth Weight Management Research Among African American Women | Study not carried out on overweight and obese individuals only |
| Protocol for a 1-year prospective, longitudinal cohort study of patients undergoing Roux-en-Y gastric bypass and sleeve gastrectomy: the BARI-LIFESTYLE observational study | Protocol |
| Weight loss intervention for obese older women: improvements in performance and function | Wearable technology used only as a means of measurement |
| Analysis of the information quality of korean obesity-management smartphone applications | No intervention carried out |
| Physical activity and quality of life in severely obese adults during a two-year lifestyle intervention programme | Wearable technology used only as a means of measurement |
| Cardiovascular health correlates of physical activity and sedentary behavior patterns in American adults | Wearable technology used only as a means of measurement |
| The use of e-health and m-health tools in health promotion and primary prevention among older adults: a systematic literature review | No emphasis on specific features of wearable technology |
| Translating a heart disease lifestyle intervention for use in south Asian immigrant communities: Preliminary results of a pilot randomized controlled trial | Not focused on weight management |
| A school-based intervention program in promoting leisure-time physical activity: trial protocol | Protocol |
| Home environments, physical activity, and energy expenditure among low-income overweight and obese women | Wearable technology used only as a means of measurement |
| Pregnancy, exercise and nutrition research study with smart phone app support (Pears): Study protocol of a randomized controlled trial | Protocol |
| Sleep and eating behavior in adults at risk for type 2 diabetes | Wearable technology used only as a means of measurement |
| Lifestyle-based physical activity intervention for one year improves metabolic syndrome in overweight male employees | Focus was on PA and not weight loss |
| Adiposity and grip strength as long-Term predictors of objectively measured physical activity in 93 015 adults: The UK Biobank study | Wearable technology used only as a means of measurement |
| Aging in neighborhoods differing in walkability and income: associations with physical activity and obesity in older adults | Demographic study |
| Insufficient and poor sleep are associated with barriers to healthy eating and lower physical activity: Baseline characteristics of the empower study | Wearable technology used only as a means of measurement |
| Body fatness, physical activity, and nutritional behaviours in Asian Indian immigrants to New Zealand | Demographic study |
| A pilot walking program promotes moderate-intensity physical activity during pregnancy | Wearable technology used only as a means of measurement |
| Objectively measured physical activity in overweight and obese pregnant women can be predicted by self-efficacy and pre-pregnancy BMI | Wearable technology used only as a means of measurement |
| A community health worker-led lifestyle behavior intervention for Latina (Hispanic) women: feasibility and outcomes of a randomized controlled trial | Study not carried out on overweight and obese individuals only |
| A Blended Web-Based Gaming Intervention on Changes in Physical Activity for Overweight and Obese Employees: Influence and Usage in an Experimental Pilot Study | Web-based intervention |
| Review of multimodal therapies for obesity treatment: Including dietary, counseling strategies, and pharmacologic interventions | Wearable technology isn’t the focus of the intervention |
| Prediabetes and type 2 diabetes are associated with increased content of dipeptide carnosine in human skeletal muscle | Not focused on weight management |
| Specific changes in plasma lipidome in obesity and type 2 diabetes are associated with physical inactivity and muscle mitochondrial function | Wearable technology used only as a means of measurement |
| Skeletal muscle mitochondrial cytochrome c oxidase activity is reduced in obese prediabetic individuals | Not focused on weight management |
| Physical inactivity and muscle mitochondrial function in diabetes are associated with specific changes in plasma lipidome | Wearable technology used only as a means of measurement |
| Pilot and Feasibility Test of a Mobile Health-Supported Behavioral Counseling Intervention for Weight Management Among Breast Cancer Survivors | Wearable technology isn’t the focus of the intervention |
| Go!: results from a quasi-experimental obesity prevention trial with hospital employees | Wearable technology isn’t the focus of the intervention |
| Physical activity is unrelated to cognitive performance in pre-bariatric surgery patients | Wearable technology used only as a means of measurement |
| Use of a mobile social networking intervention for weight management: a mixed-methods study protocol | Protocol |
| Use of a mobile social networking intervention for weight management: a mixed-methods study protocol | Duplicate |
| Adhesion to regular physical activity: The short-term role of the accelerometer | Focus was on PA and not weight loss |
| Telephone-delivered physical activity intervention for individuals with serious mental illness: a feasibility study | Telephone-based |
| Multiple measures of physical activity, dietary habits and weight status in African American and Hispanic or Latina women | Wearable technology used only as a means of measurement |
| Mediating effects of group cohesion on physical activity and diet in women of color: health is power | Wearable technology isn’t the focus of the intervention |
| Validity of the global physical activity questionnaire in the National Health Survey-Chile 2009-10 | Demographic study |
| Importance of Pain Acceptance in Relation to Headache Disability and Pain Interference in Women With Migraine and Overweight/Obesity | Not focused on weight management |
| Adapting a database of text messages to a mobile-based weight loss program: the case of the middle East | No intervention carried out |
| Comparison of Different Physical Activity Measurement Methods in Adults Aged 45 to 64 Years under Free-Living Conditions | Wearable technology used only as a means of measurement |
| Is increased physical activity associated with decreased fat and alcohol consumption among overweight and obese african american and hispanic-latina women? | Wearable technology used only as a means of measurement |
| Comparison between two methods to estimate physical activity in obese women: accelerometry and self-administered questionnaire | Wearable technology used only as a means of measurement |
| Objectively measured physical activity among US cancer survivors: considerations by weight status | Wearable technology used only as a means of measurement |
| Early results from a phone-and web-based weight management program offered through worksites | Web-based intervention |
| Physical activity improvement after surgery in morbid obese patients. measurement with objective methods. exercise and bariatric surgery | Wearable technology used only as a means of measurement |
| Protocol for the modeling the epidemiologic transition study: a longitudinal observational study of energy balance and change in body weight, diabetes and cardiovascular disease risk | Protocol |
| A mixed ecologic-cohort comparison of physical activity & weight among young adults from five populations of African origin | Wearable technology used only as a means of measurement |
| Objectively measured physical activity and sedentary time of breast cancer survivors, and associations with adiposity: findings from NHANES (2003-2006) | Wearable technology used only as a means of measurement |
| Novel Approaches to Obesity Prevention: Effects of Game Enjoyment and Game Type on Energy Expenditure in Active Video Games | Video games, not on mobile phone |
| Behavioral weight loss and physical activity intervention in obese adults with asthma. A randomized trial | Wearable technology used only as a means of measurement |
| Translating the Diabetes Prevention Program Lifestyle Intervention for Weight Loss into Primary Care: A Randomized Trial | Wearable technology isn’t the focus of the intervention |
| Effect of daily energy intake and amount of walking on body weight reduction and its maintenance | Wearable technology used only as a means of measurement |
| The independent and combined associations of physical activity and sedentary behavior with obesity in adults: NHANES 2003-06 | Wearable technology used only as a means of measurement |
| Individual, social and environmental correlates of physical activity in overweight and obese African American and Hispanic women: A structural equation model analysis | Wearable technology used only as a means of measurement |
| Behavior change intervention for patients with abdominal obesity | Wearable technology isn’t the focus of the intervention |
| Utility differences in RA and knee OA patients | Not focused on weight management |
| Structured education programme for women with polycystic ovary syndrome: A randomised controlled trial | Not focused on weight management |
| Comparison of different methods for estimating resting energy expenditure in obese adults | Accuracy assessment of wearable technology |
| Efficacy and educational role of a daily employment of the accelerometer to improve the lifestyle in overweight hypertensive population | Wearable technology used only as a means of measurement |
| Drug therapy for obstructive sleep apnoea in adults | Wearable technology used only as a means of measurement |
| Access to a behavioral weight loss website with or without group sessions increased weight loss in statewide campaign | Wearable technology isn’t the focus of the intervention |
| The influence of physical activity-induced energy expenditure on the variance in body weight change among individuals during a diet intervention | Wearable technology used only as a means of measurement |
| Study protocol for the 'HelpMeDoIt!' randomised controlled feasibility trial: an app, web and social support-based weight loss intervention for adults with obesity | Protocol |
| Usage and Dose Response of a Mobile Acceptance and Commitment Therapy App: Secondary Analysis of the Intervention Arm of a Randomized Controlled Trial | Not focused on weight management |
| Personal health technologies in employee health promotion: usage activity, usefulness, and health-related outcomes in a 1-year randomized controlled trial | Duplicate |
| The gamification of risk: how health apps foster self-confidence and why this is not enough | Duplicate |
| The gamification of risk: how health apps foster self-confidence and why this is not enough | Duplicate |
| Postnatal lifestyle intervention for overweight women with previous gestational diabetes mellitus (PAIGE): A pilot randomised controlled trial | Wearable technology isn’t the focus of the intervention |
| Healthy Weight in Lesbian and Bisexual Women Aged 40 and Older: An Effective Intervention in 10 Cities Using Tailored Approaches | Wearable technology used only as a means of measurement |
| Evaluating mobile phone applications for health behaviour change: A systematic review | Not focused on weight management |
| Impact of interviewer's body mass index on underreporting energy intake in overweight and obese women | Not focused on weight management |
| Sugar-sweetened beverage consumption in young adults: Understanding eating occasion contextual factors | Not focused on weight management |
| Physical activity of relatively high intensity in mid-pregnancy predicts lower glucose tolerance levels | Wearable technology used only as a means of measurement |
| Pedometer walking plus motivational interviewing program for Thai schizophrenic patients with obesity or overweight: a 12-week, randomized, controlled trial | Wearable technology isn’t the focus of the intervention |
| Design and focus group evaluation of a bed-integrated weight measurement system for wheelchair users | Wearable technology isn’t the focus of the intervention |
| Attitudes and barriers to physical activity and levels of physical activity in adults with obesity consuming very low calorie diets | Wearable technology used only as a means of measurement |
| Early Experience with Customized, Meal-Triggered Gastric Electrical Stimulation in Obese Patients | Wearable technology isn’t the focus of the intervention |
| Non-alcoholic fatty liver disease (NAFLD) with type 2 diabetes (T2D) have the lowest level of physical activity | Wearable technology used only as a means of measurement |
| Non-alcoholic fatty liver disease (NAFLD) is associated with low level of physical activity: A population-based study | Wearable technology used only as a means of measurement |
| Daily walking activity among male office workers in a rural town in northern Japan | Wearable technology used only as a means of measurement |
| Effects of pedometer-based physical activity intervention on abdominal fat and blood pressure: Saku communitybased randomized crossover intervention study | No emphasis on specific features of wearable technology |
| The effect of nutrition counselling and self-help resources on body weight and quality of life in overweight men treated for prostate cancer: The PROstate cancer weight MANagement (PRO-MAN) trial | Wearable technology isn’t the focus of the intervention |
| The effect of nutrition counselling and self-help resources on body weight and quality of life in overweight men treated for prostate cancer: the prostate cancer weight management (PRO-MAN) trial | Duplicate |
| Associations between program outcomes and adherence to Social Cognitive Theory tasks: process evaluation of the SHED-IT community weight loss trial for men | Duplicate |
| Does the effect of a physical activity behavioral intervention vary by characteristics of people with multiple sclerosis? | Wearable technology used only as a means of measurement |
| Initial Validation of the Activity Choice Index Among Overweight Women | Demographic study |
| Iterative development of Vegethon: a theory-based mobile app intervention to increase vegetable consumption | Duplicate |
| Mobile Technology for Vegetable Consumption: A Randomized Controlled Pilot Study in Overweight Adults | Duplicate |
| CRIB--the use of cardiac rehabilitation services to aid the recovery of patients with bowel cancer: a pilot randomised controlled trial (RCT) with embedded feasibility study | No emphasis on specific features of wearable technology |
| CRIB--the use of cardiac rehabilitation services to aid the recovery of patients with bowel cancer: a pilot randomised controlled trial (RCT) with embedded feasibility study | Duplicate |
| The effect of home exercise on ovulation induction using clomiphene citrate in overweight underserved women with polycystic ovarian syndrome | Not focused on weight management |
| Feasibility of popular m-health technologies for activity tracking among individuals with serious mental illness | Accuracy assessment of wearable technology |
| Feasibility of popular m-health technologies for activity tracking among individuals with serious mental illness | Duplicate |
| The cross-sectional association between snacking behaviour and measures of adiposity: the Fenland Study, UK | Wearable technology used only as a means of measurement |
| The effect of vitamin D supplementation on physical performance and activity in non-western immigrants | Wearable technology used only as a means of measurement |
| The effect of vitamin D supplementation on physical performance and activity in non-western immigrants | Duplicate |
| The effect of vitamin D supplementation on physical performance and activity in non-western immigrants | Duplicate |
| Effect of resistance exercise training on body composition and physical ftness after gastric bypass (PROMISE study) - A randomized controlled trial | Wearable technology used only as a means of measurement |
| Profile of physical activity behaviors among Swedish women aged 56-75 years | Wearable technology used only as a means of measurement |
| The steps to health employee weight management randomized control trial: rationale, design and baseline characteristics | No intervention carried out |
| The steps to health employee weight management randomized control trial: rationale, design and baseline characteristics | Duplicate |
| Self-reported and accelerometer-measured physical activity by body mass index in US Hispanic/Latino adults: HCHS/SOL | Wearable technology used only as a means of measurement |
| Pedometer measurement of physical activity and chronic disease risk factors of obese lower socioeconomic status African American women | Wearable technology used only as a means of measurement |
| Relation of body composition to daily physical activity in free-living Japanese adult women | Wearable technology used only as a means of measurement |
| The effect of obesity on exercise capacity, activity levels and health related quality of life in patients with chronic obstructive pulmonary disease | Wearable technology used only as a means of measurement |
| Effects of a Weight Loss Program on Metabolic Syndrome, Eating Disorders and Psychological Outcomes: Mediation by Endocannabinoids? | Wearable technology isn’t the focus of the intervention |
| Associations among physical activity, diet quality, and weight status in US adults | Demographic study |
| Design and Implementation of a Randomized Controlled Social and Mobile Weight Loss Trial for Young Adults (project SMART) | Web-based |
| A cross-sectional study of geographic differences in health risk factors among young Australian adults: the role of socioeconomic position | Demographic study |
| A 24-week dietary and physical activity intervention leads to sustained improvements in body mass index and insulin resistance in the obese with insulin resistant chronic hepatitis C | Wearable technology isn’t the focus of the intervention |
| Criterion validity of the Physical Activity Scale (PAS2) in Danish adults | Wearable technology used only as a means of measurement |
| Optimization of Remotely Delivered Intensive Lifestyle Treatment for Obesity using the Multiphase Optimization Strategy: Opt-IN Study Protocol | Protocol |
| Preferences and motivation for weight loss among knee replacement patients: implications for a patient-centered weight loss intervention | Wearable technology isn’t the focus of the intervention |
| Preferences and motivation for weight loss among knee replacement patients: implications for a patient-centered weight loss intervention | Duplicate |
| A customized low glycaemic-index (GI) diet prevents both the gestational diabetes mellitus (GDM) and the large for gestational age (LGA) babies in overweight/obese pregnant women | Wearable technology isn’t the focus of the intervention |
| Modeling individual differences: A case study of the application of system identification for personalizing a physical activity intervention | Wearable technology isn’t the focus of the intervention |
| Modeling individual differences: A case study of the application of system identification for personalizing a physical activity intervention | Wearable technology isn’t the focus of the intervention |
| Self-report versus objective measures of physical activity in overweight/obese latina immigrants in alabama | Wearable technology used only as a means of measurement |
| Pilot and Feasibility Test of a Mobile Health-Supported Behavioral Counseling Intervention for Weight Management Among Breast Cancer Survivors | Text messaging |
| Accelerometer thresholds: Accounting for body mass reduces discrepancies between measures of physical activity for individuals with overweight and obesity | Accuracy assessment of wearable technology |
| Predicted vs. Actual Resting Energy Expenditure and Activity Coefficients: Post-Gastric Bypass, Lean and Obese Women | Wearable technology used only as a means of measurement |
| Ideal cardiovascular health (ICVH) in patients with a recent diagnosis of colorectal cancer (CRC) | Not focused on weight management |
| Adherence to physical activity guidelines and its relationship with self-rated health among persons with doctor-diagnosed arthritis | Wearable technology used only as a means of measurement |
| Long-term health benefits of physical activity – a systematic review of longitudinal studies | Wearable technology used only as a means of measurement |
| Pathways through which higher neighborhood crime is longitudinally associated with greater body mass index | Not a weight loss intervention |
| One size doesn’t fit all: cross-sectional associations between neighborhood walkability, crime and physical activity depends on age and sex of residents | Not a weight loss intervention |
| Feasibility of adding enhanced pedometer feedback to nutritional counseling for weight loss | Wearable technology isn’t the focus of the intervention |
| An online community improves adherence in an internet-mediated walking program. Part 1: results of a randomized controlled trial | Web-based |
| Implementation of interval walking training in patients with type 2 diabetes in Denmark: Rationale, design, and baseline characteristics | Demographic study |
| Validating stage of change measures for physical activity and dietary behaviors for overweight women | Accuracy assessment of wearable technology |
| Study of a community intervention program on physical activity in obese and overweight patients | Wearable technology isn’t the focus of the intervention |
| The Feasibility of Reducing Sitting Time in Overweight and Obese Older Adults | Wearable technology used only as a means of measurement |
| Reliability and validity of the Sedentary Behavior Questionnaire (SBQ) for adults | Wearable technology used only as a means of measurement |
| Tailored weight loss program for patients with moderate to severe COPD | Wearable technology isn’t the focus of the intervention |
| Functionalities and input methods for recording food intake: A systematic review | Not a weight loss intervention |
| Are women with obesity and infertility willing to attempt weight loss prior to fertility treatment? | No intervention carried out |
| Effective behaviour change techniques for physical activity and healthy eating in overweight and obese adults; systematic review and meta-regression analyses | Wearable technology isn’t the focus of the intervention |
| Effective behaviour change techniques for physical activity and healthy eating in overweight and obese adults; systematic review and meta-regression analyses | Duplicate |
| Cultural Variables Underlying Obesity in Latino Men: Design, Rationale and Participant Characteristics from the Latino Men's Health Initiative | No intervention carried out |
| A population-based lifestyle intervention to promote healthy weight and physical activity in people with cardiac disease: the PANACHE (Physical Activity, Nutrition And Cardiac HEalth) study protocol | Protocol |
| Telemedical assessment of the level of energy expenditure in overweight and obese individuals | Telephone-based |
| Weight Management to Reduce Prostate Cancer Risk: A Survey of Men's Needs and Interests | No intervention carried out |
| Influence of Individual Determinants on Physical Activity at Work and During Leisure Time in Soldiers: A Prospective Surveillance Study | Focus was on PA and not weight loss |
| Both body mass index and waist circumference are adversely associated with exposure to trans-fatty acids in persons with type 2 diabetes | Wearable technology used only as a means of measurement |
| Plasma fatty acid concentrations vary seasonally in persons with type 2 diabetes | Wearable technology used only as a means of measurement |
| Adverse plasma fatty acid composition in persons with type 2 diabetes who use statins may contribute to residual cardiovascular risk | Wearable technology used only as a means of measurement |
| Devices for ambulatory and home monitoring of blood pressure, lipids, coagulation, and weight management, part 1 | Not a weight loss intervention |
| Relationship between accelerometer-based measures of physical activity and the yale physical activity survey in adults with arthritis | Wearable technology used only as a means of measurement |
| Effective modification of eating behavior and weight loss using abilitiTM system in obese subjects | Wearable technology used only as a means of measurement |
| Text4Diet: a randomized controlled study using text messaging for weight loss behaviors | Text messaging |
| Design and validation of a low cost, high-capacity weighing device for wheelchair users and bariatrics | Not a weight loss intervention |
| Design and baseline characteristics of participants in the Enhancing Physical Activity and Reducing Obesity through Smartcare and Financial Incentives | No intervention carried out |
| Resting metabolic rate, cardiorespiratory fitness, and racial differences in adiposity among adult women | Wearable technology used only as a means of measurement |
| Electronic feedback in a diet- and physical activity-based lifestyle intervention for weight loss: a randomized controlled trial | Duplicate |
| Neighbourhood environment correlates of physical activity: A study of eight czech regional towns | Demographic study |
| Assessing physical inactivity using accelerometers in comparison to international physical activity questionnaire | Wearable technology used only as a means of measurement |
| The feasibility phase of a community antenatal lifestyle programme [The Lifestyle Course (TLC)] for women with a body mass index (BMI)>= 30 kg/m(2.) | Wearable technology isn’t the focus of the intervention |
| The feasibility phase of a community antenatal lifestyle programme [The Lifestyle Course (TLC)] for women with a body mass index (BMI)>= 30 kg/m(2.) | Duplicate |
| Cognitive remediation therapy plus behavioural weight loss compared to behavioural weight loss alone for obesity: study protocol for a randomised controlled trial | Protocol |
| Effects of Body Mass Index on Bone Loading Due to Physical Activity | Focus was on PA and not weight loss |
| Does physical activity influence the relationship between low back pain and obesity? | Wearable technology used only as a means of measurement |
| Does physical activity influence the relationship between low back pain and obesity? | Duplicate |
| Does physical activity influence the relationship between low back pain and obesity? | Duplicate |
| Maternal Obesity Management Using Mobile Technology: A Feasibility Study to Evaluate a Text Messaging Based Complex Intervention during Pregnancy | Text messaging |
| Interventions for supporting the initiation and continuation of breastfeeding among women who are overweight or obese | Not a weight loss intervention |
| Effects of an Abbreviated Obesity Intervention Supported by Mobile Technology: The ENGAGED Randomized Clinical Trial | Wearable technology isn’t the focus of the intervention |
| Social support for physical activity and healthy eating in a worksite weight management program for obese workers | Wearable technology isn’t the focus of the intervention |
| Type 1 diabetes patients eating behaviour analysis-alarming results | Wearable technology isn’t the focus of the intervention |
| Positive effect of the use of accelerometry on lifestyle awareness of overweight hypertensive patients | No user experience component |
| Mobile phone interventions to increase physical activity and reduce weight: a systematic review | Text messaging |
| Problems in identifying predictors and correlates of weight loss and maintenance: Implications for weight control therapies based on behaviour change | Wearable technology isn’t the focus of the intervention |
| Ambulatory activity and body mass index in white and non-white older adults | Wearable technology used only as a means of measurement |
| Physical Activity Patterns of Latina Immigrants Living in Alabama | Wearable technology used only as a means of measurement |
| Assessment of physical activity in hypertrophic cardiomyopathy | Wearable technology used only as a means of measurement |
| Effect of pedometer use and goal setting on walking and functional status in overweight adults with multimorbidity: A crossover clinical trial | No user experience component |
| Optimising healthy gestational weight gain in women at high risk of gestational diabetes: A randomised controlled trial | Wearable technology used only as a means of measurement |
| Validity and reliability of a physical activity recall instrument among overweight and non-overweight men and women | Wearable technology used only as a means of measurement |
| Predictors of community walking participation and walking capacity in people with lumbar spinal stenosis | Wearable technology used only as a means of measurement |
| The spinal stenosis pedometer and nutrition lifestyle intervention (SSPANLI) pilot study | Web-based intervention |
| The spinal stenosis pedometer and nutrition lifestyle intervention (SSPANLI) randomized controlled trial protocol | Protocol |
| The spinal stenosis pedometer and nutrition lifestyle intervention (SSPANLI): development and pilot | Web-based intervention |
| The spinal stenosis pedometer and nutrition lifestyle intervention (SSPANLI) randomized controlled trial protocol | Duplicate |
| Midwifery E-Health: From design to validation of "mammastyle - Gravidanza Fisiologica" | Web-based intervention |
| Accelerometer profiles of physical activity and inactivity in normal weight, overweight, and obese U.S. men and women | Demographic study |
| The Dietary Intervention to Enhance Tracking with Mobile Devices (DIET Mobile) Study: A 6-Month Randomized Weight Loss Trial | Duplicate |
| Weight-supported (anti-gravity) treadmill exercise in caribbean-black obese women | Wearable technology isn’t the focus of the intervention |
| Effectiveness of a worksite mindfulness-based multi-component intervention on lifestyle behaviors | Wearable technology isn’t the focus of the intervention |
| Effectiveness of a worksite mindfulness-based multi-component intervention on lifestyle behaviors | Duplicate |
| The intervention process in the European Fans in Training (EuroFIT) trial: a mixed method protocol for evaluation | Protocol |
| A pilot intervention to reduce postpartum weight retention and central adiposity in first-time mothers: results from the mums OnLiNE (Online, Lifestyle, Nutrition & Exercise) study | Wearable technology isn’t the focus of the intervention |
| A pilot intervention to reduce postpartum weight retention and central adiposity in first-time mothers: results from the mums OnLiNE (Online, Lifestyle, Nutrition & Exercise) study | Duplicate |
| Reliability and validity of the international physical activity questionnaire for assessing walking | Wearable technology used only as a means of measurement |
| International study of objectively measured physical activity and sedentary time with body mass index and obesity: IPEN adult study | Wearable technology used only as a means of measurement |
| Cross-sectional surveillance study to phenotype lorry drivers' sedentary behaviours, physical activity and cardio-metabolic health | Wearable technology used only as a means of measurement |
| A pedometer-based walking intervention with and without email counseling in general practice: a pilot randomized controlled trial | Email-based intervention |
| Effectiveness of cognitive behavioral therapy on changes of anthropometric and biochemical parameters in group weight reduction courses | Wearable technology isn’t the focus of the intervention |
| A comparison of physical activity, physical fitness levels, BMI and blood pressure of adults with intellectual disability, who do and do not take part in Special Olympics Ireland programmes: Results from the SOPHIE study | Wearable technology used only as a means of measurement |
| Objective physical activity and quality of life in breast and colon cancer patients after completion of adjuvant chemotherapy | Wearable technology used only as a means of measurement |
| Associations between self-reported and objectively measured physical activity, sedentary behavior and overweight/obesity in NHANES 2003-2006 | Wearable technology used only as a means of measurement |
| Safety of strength training in premenopausal women: musculoskeletal injuries from a two-year randomized trial | Wearable technology used only as a means of measurement |
| Comparison of leisure physical activity and exercise capacity in women with fibromyalgia and healthy matched controls | Wearable technology used only as a means of measurement |
| Depressive symptoms are associated with dietary intake but not physical activity among overweight and obese women from disadvantaged neighborhoods | Wearable technology used only as a means of measurement |
| Interactive computer-based interventions for weight loss or weight maintenance in overweight or obese people | Web-based intervention |
| Guide to Health: A Randomized Controlled Trial of the Effects of a Completely WEB-Based Intervention on Physical Activity, Fruit and Vegetable Consumption, and Body Weight | Web-based intervention |
| Daily Text Messaging for Weight Control Among Racial and Ethnic Minority Women: Randomized Controlled Pilot Study | Text messaging |
| Physical activity of daily living in patients with schizophrenia | Wearable technology used only as a means of measurement |
| Effectiveness of a pragmatic education program designed to promote walking activity in individuals with impaired glucose tolerance: a randomized controlled trial | Wearable technology used only as a means of measurement |
| The effectiveness of computerized services and peer coaches to improve weight in patients with serious mental illness | Wearable technology isn’t the focus of the intervention |
| Effect of a Gender-Tailored eHealth Weight Loss Program on the Depressive Symptoms of Overweight and Obese Men: Pre-Post Study | Not focused on weight management |
| Association of fitbit activity monitor and human activity profile with clinical markers of health and fitness | Wearable technology used only as a means of measurement |
| A novel prescription pedometer-assisted walking intervention and weight management for Chinese occupational population | Study not carried out on overweight and obese individuals only |
| Acceptability and feasibility of smartphone–assisted twenty-four-hour recalls in the Chinese population | Study not carried out on overweight and obese individuals only |
| Nutrition and physical activity in NAFLD: An overview of the epidemiological evidence | Focus was on PA and not weight loss |
| Interactions between physical activity and risk factors of osteoarthritis on mri-detected osteophytes in a population based cohort study | Focus was on PA and not weight loss |
| Interactions between physical activity and risk factors of osteoarthritis on mri-detected osteophytes in a population based cohort study | Duplicate |
| Online and smartphone based cognitive behavioral therapy for bariatric surgery patients: Initial pilot study | Study not carried out on overweight and obese individuals only |

**Manually excluded after full-text review**

| **Study title** | **Reason for exclusion** |
| --- | --- |
| Randomized Controlled Pilot Study Testing Use of Smartphone Technology for Obesity Treatment | Lack of focus on specific features of wearable technology. Results shows the effect of smartphone technology but not which feature may have achieved such effect. |
| Relationship Between Evidence Requirements, User Expectations, and Actual Experiences: Usability Evaluation of the Twazon Arabic Weight Loss App | Lack of appropriate measurement, such as weight reduction. Intervention carried out for too short a duration for any effect to be observed. |
| Toward Health Information Technology that Supports Overweight and Obese Women in Addressing Emotion- and Stress-Related Eating (a Mixed Methods Approach) | Lack of appropriate measurement, and the aim of the intervention is biased towards the emotional aspect. Weight change is somehow overlooked |
| What Overweight Women Want From a Weight Loss App: A Qualitative Study on Arabic Women | No intervention carried out, only a focus-group discussion of studies to be included. Not backed up with evidence |
| A Serious Game to Increase Healthy Food Consumption in Overweight or Obese Adults: Randomized Controlled Trial | Lack of appropriate measurement, the study outcome is somehow vague and not directly associated with weight loss. |
| Behavioral Mediators of Treatment Effects in the Weight Loss Maintenance Trial | Lack of focus on specific features of wearable technology, only talked about the general aspect |
| Development of a Smartphone Application for Clinical-Guideline-Based Obesity Management | No intervention carried out. A lot of theory-based approach but no data collected yet to back them up. |
| Factors Affecting Acceptance of Smartphone Application for Management of Obesity | Intervention not carried out on overweight/obese individuals |
| myPace: an integrative health platform for supporting weight loss and maintenance behaviors | No intervention carried out. A lot of theory-based approach but no data collected yet to back them up. |
| The effects of acceptance and commitment therapy on eating behavior and diet delivered through face-to-face contact and a mobile app/ a randomized controlled trial | Mobile app used only as a means of intervention delivery, the main focus is on the effect of acceptance and commitment therapy. |
| An online community improves adherence in an internet-mediated walking program. Part 1/ results of a randomized controlled trial | Web-based intervention |
| BMI is Associated with the Willingness to Record Diet with a Mobile Food Record among Adults Participating in Dietary Interventions | Intervention not carried out on only overweight/obese individuals |
| Development of a Healthy Lifestyle Mobile App for Overweight Pregnant Women/ Qualitative Study | No intervention carried out. A lot of theory-based approach but no data collected yet to back them up. |
| Effect of a Gender-Tailored eHealth Weight Loss Program on the Depressive Symptoms of Overweight and Obese Men/ Pre-Post Study | Lack of focus on specific features of wearable technology. Also the focus is on the depressive symptoms and not weight loss. |
| Personal health technologies in employee health promotion/ Usage activity, usefulness, and health-related outcomes in a 1-year randomized controlled trial | Intervention not carried out on only overweight/obese individuals |
| The design and conduct of Keep It Off/ An online randomized trial of financial incentives for weight loss maintenance | Lack of focus on specific features of wearable technology. Method of delivery mainly online. |
| An evidence-based gamified mHealth intervention for overweight young adults with maladaptive eating habits/ Study protocol for a randomized controlled trial | Only a protocol |
| Understanding messaging preferences to inform development of mobile goal-directed behavioral interventions | Intervention not carried out on overweight/obese individuals. |
